# Supplementary material for: The Curious Case of A31P, a Topology-Switching Mutant of the Repressor of Primer Protein: A Molecular Dynamics Study of Its Folding and Misfolding
Source: J Chem Inf Model. 2024 Jul 25;64(15):6081–91. doi: 10.1021/acs.jcim.4c00575 (PMC11323272; doi:10.1021/acs.jcim.4c00575)
Supplement: Supplementary file 1 — ci4c00575_si_001.pdf [file ci4c00575_si_001.pdf]

Supplementary Information File

The curious case of A31P, a topology-switching mutant of the Repressor of Primer protein : A molecular dynamics study of its folding and misfolding

Olympia-Dialekti Vouzina, Alexandros Tafanidis

& Nicholas M. Glykos*

Department of Molecular Biology and Genetics, Democritus University of Thrace, University campus, 68100 Alexandroupolis, Greece, Tel +30-25510-30620, Fax +30-25510-30620, https://utopia.duth.gr/glykos/ , glykos@mbg.duth.gr

Figure S1

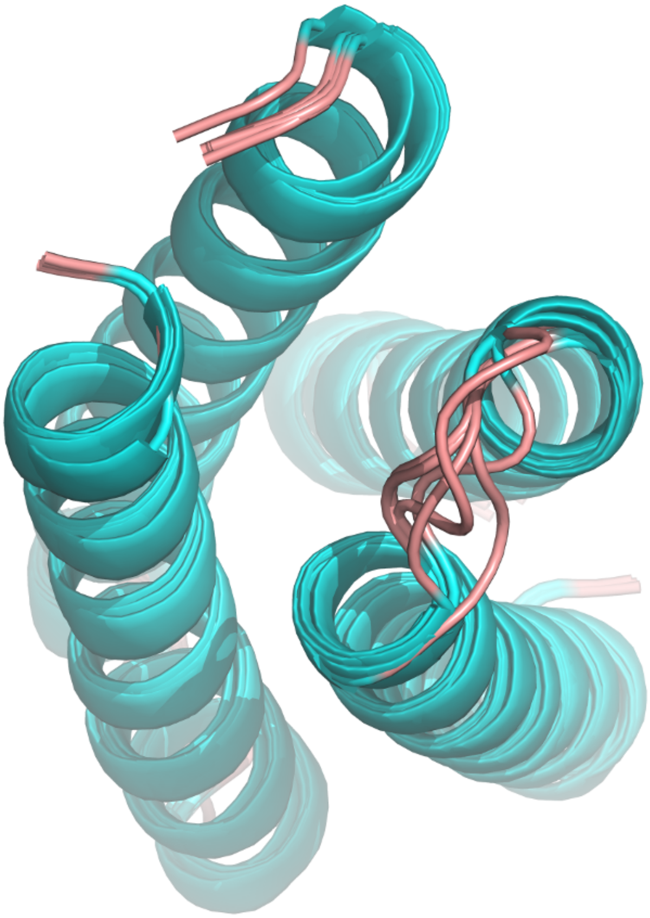

Fig.S1 Superposition of all mutant structures modelled with AlphaFold : Least squares superposition of all structures prepared with AlphaFold-multimer including the experimentally observed native Rop structure. The corresponding RMSDs are shown in Table I of the main text, and as can clearly be seen from this image, all predicted structures (including A31P) are essentially indistinguishable, both inbetween them, and also with the crystallographically determined native Rop structure. The loop region that appears to deviate the most from the rest (rightmost in this figure), corresponds to the 2AA insertion mutant.

Figure S2

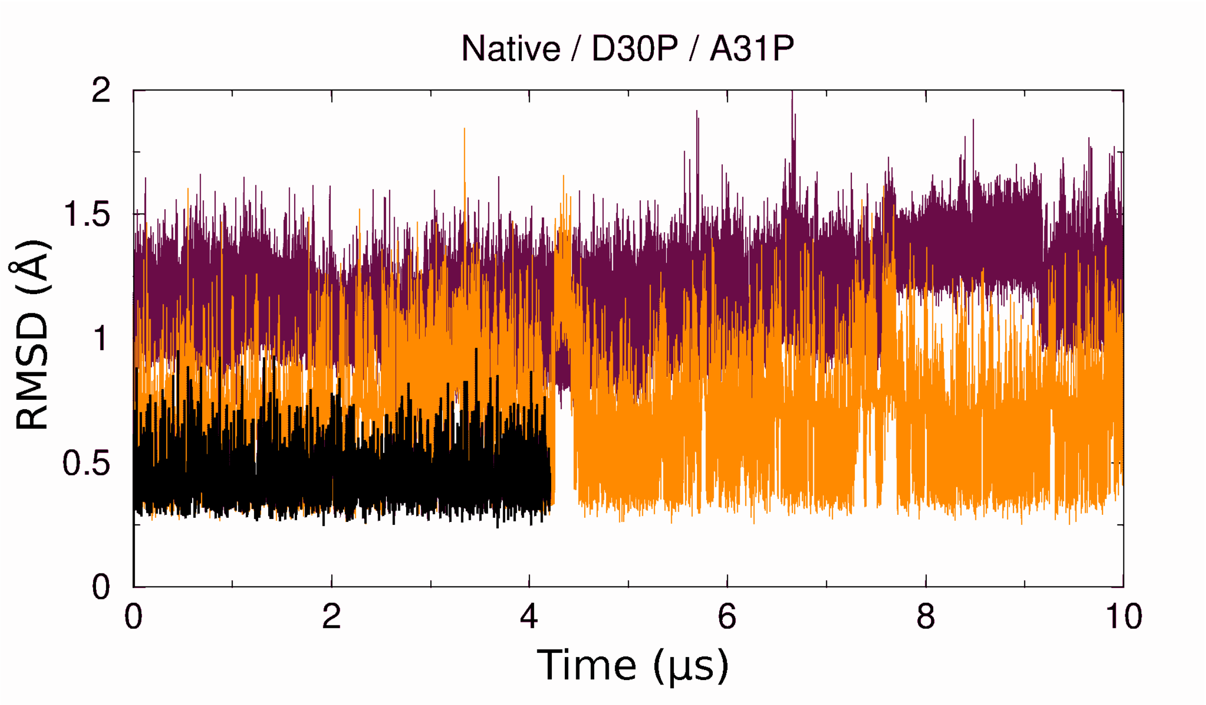

Fig.S2 Comparison of A31P vs. D30P vs. Native Rop : This diagram compares the RMSDs from the initial structures for native-like-A31P (upper magenta curve), the D30P mutant (orange curve), and native Rop (lower black curve) as a function of simulation time (in μs). The RMSDs were calculated using the Cα atoms of the turn residues only (24-39 inclusive) of the respective proteins. These results were obtained from three independently performed simulations using the program gromacs, the same force field (AMBER99SB-STAR-ILDN), the same initial structures, and the same simulation conditions (*NpT* at 320K) as discussed in the main text. The behavior of the native Rop simulation was essentially identical with the one shown in Figures 5 & 6 of the main manuscript, and the simulation was discontinued after 4 μs.

Notice how for this set of simulations an outstanding unfolding event for native-like-A31P has not been observed (compare with Fig.6 of the main paper), emphasizing the stochastic nature of the initiation of unfolding as discussed in sections 3.5 and 4 of the main manuscript. Having said that, native-like-A31P appears to be the least stable of the three structures studied here, with RMSDs consistently higher than the other two proteins.

The D30P mutant (which is known from the experiment to maintain a native fold and topology[1]) does show a native-like behaviour, but with notable excursions to higher RMSDs. The apparent instability of D30P compared with the native Rop is in good agreement with the thermodynamic data known for this mutant : D30P has a melting temperature of 58.9°C , significantly lower than the 68.7°C measured for native Rop in the same set of experiments.[1] Considering that these *NpT* simulations have been performed at a mean temperature of 46.9°C , it appears that the simulations correctly reproduce the expected difference in stability and structural fluctuations of two otherwise stably folded proteins but with Tm's of 58.9 vs. 68.7°C respectively.

References

(1) Predki, P. F.; Agrawal, V.; Brünger, A. T.; Regan, L. Amino-Acid Substitutions in a Surface Turn Modulate Protein Stability. *Nat. Struct. Biol.* **1996**, *3*, 54–58. https://doi.org/10.1038/nsb0196-54.
